# The effect of dose timing schedule on live performance, carcass cutting yields, and fat quality of market gilts managed using GnRF immunization

**DOI:** 10.1093/tas/txag043

**Published:** 2026-04-06

**Authors:** B M Bohrer, B Hansen, D S Pollmann, K A Vonnahme, L Van De Weyer, A Aldaz

**Affiliations:** Department of Animal Sciences, The Ohio State University, Columbus, OH, 43210, United States; BCH Consulting LLC, Atlantic, IA, 50022, United States; DSP Consulting LLC, Alpine, UT, 84004, United States; Zoetis Inc., Parsippany, NJ, 07054, United States; Zoetis Canada Inc, Kirkland, Quebec, H9H 4M7, Canada; Zoetis Inc., Parsippany, NJ, 07054, United States

**Keywords:** anti-GnRF, estrous suppression, ovarian activity, carcass cutting yield, meat quality, fat deposition

## Abstract

The objective of this study was to evaluate the effects of dose timing on live performance, carcass cutting yields, and fat quality of gilts managed using GnRF immunization. The 15-week study began when 1056 gilts (11 weeks old; average starting weight of 31.7 kg) were allocated by weight to 48 pens, with 22 gilts per pen. Experimental treatments were arranged as a 2 × 2 factorial, with main effects of the interval between dose 1 and dose 2 of the GnRF immunization (4 or 6 weeks; interval between doses) and the interval between dose 2 and marketing [weighted averages of 49 days (long) or 35 days (short); marketing interval]. Age and days on feed were consistent among treatment groups, and pigs were marketed using a similar strategy, in which the heaviest 3–5 pigs from each pen were marketed during study week 12, the next heaviest 3–5 pigs from each pen were marketed during study week 14, and the remaining pigs in each pen were marketed during study week 15. Hot carcass weight and optical probe readings were collected at slaughter. Following slaughter, 283 carcasses (target of 24 per treatment for each marketing event) were selected for evaluation of carcass cutting yields and meat quality. There were limited interactions between interval between doses and marketing interval, with nonsignificant interactions (*P* ≥ 0.07) observed for average daily feed intake (ADFI), average daily gain (ADG), and gain: feed (G: F) ratio during the grow-finish period, as well as for hot carcass weight, backfat thickness, and carcass primal weights. Overall, this study illustrated the trade-offs associated with altering the time from dose 2 to marketing. From a live production standpoint, there were significant differences (*P* < 0.01) in time from dose 2 to marketing for ADFI and feed efficiency (F: G ratio), with the short marketing interval resulting in lower ADFI (2.47 vs. 2.53 kg) and improved F: G (2.69 vs. 2.74) compared with the long marketing interval. From a meat processing standpoint, differences in fat deposition were observed for marketing interval. The long marketing interval resulted in 0.78 mm greater backfat thickness, 0.15 kg heavier trimmed bellies, a 1.30-unit lower iodine value, and firmer bellies compared with the short marketing interval. Thus, producers utilizing GnRF immunization for gilts should closely monitor the interval between dose 2 and marketing to optimize live performance and carcass quality.

## Teaser

Careful management is required to balance the trade-off between live production performance and processing suitability (ie appropriate carcass leanness and fat quality) when using GnRF immunization in gilts. Adjusting the interval between the second GnRF immunization dose and marketing can meaningfully impact live performance and fat deposition.

## Introduction

In commercial swine production systems, managing gilts during the finishing phase presents several biological and logistical challenges that can impact growth performance, carcass composition, and overall production efficiency. Unlike barrows, gilts exhibit cyclic estrous behavior during the finishing period, which can reduce feed intake consistency, increase activity, and negatively affect growth performance and feed efficiency (Almeida et al. 2000). Additionally, the onset of estrus introduces variability in carcass composition due to differences in nutrient partitioning between lean and fat deposition (Overholt et al. 2016; Bohrer et al. 2023a). These factors can ultimately contribute to greater heterogeneity in both live performance and carcass quality, creating challenges for producers aiming to optimize efficiency and product consistency.

Improvest (marketed under the trade names Improvac, Innosure, or Vivax in some parts of the world; Zoetis Inc., Parsippany, NJ, USA) is a gonadotropin-releasing factor (GnRF) analogue–diphtheria toxoid conjugate product approved for the temporary suppression of ovarian function and estrus in intact female pigs. Managing market pigs with GnRF immunization offers several secondary production advantages (Poulsen Nautrup et al. 2020). Specifically, in gilts, suppression of ovarian function can result in a 10% to 15% improvement in weight gain during the final 3 to 7 weeks of the finishing period (3% to 6% improvement when calculated across the entire grow-finish period), leading to 3 to 5 kg heavier hot carcass weights (HCW) compared with untreated (ie non-immunized) gilts (Bohrer et al. 2014; Poulsen Nautrup et al. 2020; Vasquez-Hidalgo et al. 2023).

Closely monitoring both the interval between doses and the interval between dose 2 and marketing is hypothesized to influence the production benefits of GnRF immunization in gilts. Specifically, degradation of antibody titers as the interval between doses is extended could lead to a diminished growth response. In addition, feed efficiency may decline as the interval between dose 2 and marketing is extended (Allison et al. 2021; Bohrer et al. 2024b). Therefore, the objective of this study was to evaluate the effects of dose timing on live performance, carcass cutting yields, and fat quality of gilts managed using GnRF immunization.

## Materials and methods

The experiment was conducted at a commercial research grow-finish facility located in Manitoba, Canada. Pigs were managed in accordance with the Canadian Council on Animal Care (2009).

### Animals

One thousand fifty-six gilts (progeny of commercial Duroc sires × white-line sows) were weighed and allotted to twelve replications based on weight, with each replication consisting of four treatments (*described in a subsequent section*). Within each replication, treatments were randomly assigned to one of 48 pens (22 gilts/pen; initial stocking density of 0.68 m^2^/pig) across four rooms (12 pens/room) in the commercial grow-finish barn. Pens were equipped with a single three-hole wet/dry stainless-steel feeder (Crystal Spring Hog Equipment; Ste. Agathe, Manitoba, Canada) and a single bowl drinker. Rooms were ventilated using negative pressure and maintained within thermoneutral conditions for grow-finish pigs. Pigs had ad libitum access to feed and water throughout the study.

### Experimental design

Gilts were administered Improvest (a GnRF analogue–diphtheria toxoid conjugate product; Zoetis Inc.) *via* two sequential injections (ie doses) by trained personnel according to manufacturer directions. The first dose served as a priming dose, and the second dose served as a booster that triggered temporary suppression of ovarian function and estrus.

Four dose-timing schedules were evaluated in this study (Table 1), with all pigs marketed at a similar age and days on feed. The interval between doses of the GnRF immunization was either 4 or 6 weeks, and the interval between dose 2 and marketing was either a weighted average of 49 days (long) or 35 days (short). This resulted in a 2 × 2 factorial design with two independent factors, each at two levels.

**Table 1:** Description of treatments.^a–c^

*Number or Pen Replicates*	Dose-1	Dose-2	Marketing Event #1	Marketing Event #2	Marketing Event #3	Interval Between Doses	Marketing Interval
***12***	wk-1	wk-7	wk-12	wk-14	wk-15	6 wk	Long (7-wk weighted average)
***12***	wk-3	wk-7	wk-12	wk-14	wk-15	4 wk	Long (7-wk weighted average)
***12***	wk-3	wk-9	wk-12	wk-14	wk-15	6 wk	Short (5-wk weighted average)
***12***	wk-5	wk-9	wk-12	wk-14	wk-15	4 wk	Short (5-wk weighted average)

a At the start of the study, pigs were 11 wk old with an average starting weight of 31.7 kg.

b The interval between doses of the GnRF immunization was either 4 or 6 wks and the marketing interval was either a weighted average of 49 days (Long) or a weighted average of 35 days (Short).

c Pigs were marketed in three groups on wk-12, wk-14, and wk-15 of the study; the heaviest 3–5 pigs in each pen (approximately 18%) were marketed on wk-12 and wk-14, and the barn dump (approximately 64%) occurred on wk-15.

### Diets

All treatments were fed the same diets and feeding program (Table 2). The experiment included five diet phases: Phase 1 (d 0 to 19; 32 to 50 kg), Phase 2 (d 20 to 29; 50 to 60 kg), Phase 3 (d 30 to 58; 60 to 86 kg), Phase 4 (d 59 to 84; 86 to 112 kg), and Phase 5 (d 85 to marketing; 112 to 135 kg). Diets were fed in pelleted form and formulated using Brill formulation software (Agrifood Alternative Technologies; Lleida, Spain) to meet or exceed nutrient requirements for gilts.

**Table 2:** Composition of the diets formulated for each dietary phase throughout the grow-finisher period.

	Phase 1	Phase 2	Phase 3	Phase 4	Phase 5
	(d 0–19; 32–50 kg)	(d 20–29; 50–60 kg)	(d 30–58; 60–86 kg)	(d 59–84; 86–112 kg)	(d 85—marketing; 112–135 kg)
***Ingredient, %***					
*** Wheat***	50.00	50.00	50.00	50.00	50.00
*** Corn***	22.16	28.28	30.42	21.57	14.42
*** Barley***	–	–	–	7.26	13.37
*** Soybean meal***	17.08	11.89	6.26	–	–
*** Corn DDGS***	6.66	6.49	10.94	18.45	19.58
*** Limestone***	1.17	1.12	1.07	1.56	1.60
*** Soybean oil***	1.53	0.85	–	–	–
*** Sodium chloride***	0.40	0.41	0.36	0.35	0.33
*** Vitamin/trace mineral premix^a^***	0.12	0.12	0.12	0.12	0.12
*** Phytase***	0.02	0.01	0.01	0.01	0.01
*** Lipinate^b^***	–	–	–	0.05	0.05
*** L-lysine***	0.55	0.55	0.55	0.49	0.42
*** L-threonine***	0.16	0.16	0.14	0.08	0.05
*** DL-methionine***	0.12	0.09	0.04	–	–
*** L-tryptophan***	0.03	0.03	0.03	0.01	–
***Calculated analysis^c^***					
***Crude protein, %***	19.76	17.79	16.61	15.86	16.19
***Crude fat, %***	3.87	3.33	2.85	3.23	3.19
***Crude fiber, %***	2.97	2.90	3.08	3.57	3.79
***Calcium, %***	0.64	0.60	0.56	0.76	0.78
***STTD phosphorus, %***	0.32	0.30	0.28	0.31	0.32
***SID amino acids, %***					
*** Lys***	1.11	0.99	0.87	0.70	0.65
*** Met***	0.36	0.32	0.26	0.22	0.23
*** Met + Cys***	0.65	0.58	0.51	0.46	0.47
*** Thr***	0.70	0.63	0.56	0.45	0.43
*** Trp***	0.22	0.20	0.17	0.14	0.13
*** Net energy, kcal/kg***	2475	2475	2450	2425	2400
*** SID Lys: NE, g/Mcal***	4.50	4.00	3.55	2.90	2.70

a The vitamin and trace mineral premix included 25 ppm of salinomycin sodium (Phibro Animal Health; Teaneck NJ, USA) throughout the study period.

b 500 ppm of Lipinate (Nutriquest; Mason City, IA, USA) was added to Phase 4 and Phase 5 diets.

c Composition was calculated using Brill formulation software (Agrifood Alternative Technologies; Lleida, Spain).

### Collection of on-farm data

Feed delivery was recorded daily using a precision feeding system (Gestal XM; Jyga Technologies, Saint-Lambert-de-Lauzon, Quebec, Canada). Pen weights were collected at the start of the study (d 0) and on d 21, 35, 49, 63, 85 (first marketing event), 98 (second marketing event), and 105 (final marketing event). Individual pig weights were recorded on marketing days. Gain: feed (G: F) was calculated as average daily gain (ADG) divided by average daily feed intake (ADFI). Mortalities and removals were recorded for each pen, and subsequent growth performance calculations were adjusted to reflect pigs remaining in the pen.

### Marketing and slaughter procedures

An equal number of gilts were marketed from each treatment group on d 85 (heaviest 3 to 5 gilts per pen; approximately 18% of the population), d 98 (heaviest 3 to 5 gilts per pen; approximately 18%), and d 105 (remaining 12 to 16 gilts per pen; approximately 64%). Depending on the dose-timing schedule, these marketing events corresponded to weighted averages of either 35 or 49 days between dose 2 and marketing.

Pigs were slaughtered under commercial conditions and identification was maintained throughout the slaughter process with a tattoo that reflected the pen number. The slaughter facility utilized group CO_2_ gas stunning and conventional chilling (20–24 h of exposure to ambient temperatures of approximately 2°C). Hot carcass weights were recorded by processing plant personnel and carcass dressing percentage was calculated as HCW divided by weight at marketing. Grading probe measurements (ie backfat thickness and muscle depth) were collected on-line using the left side of carcasses by experienced operators from provincial grading authorities using a Destron PG-203 probe (Anitech Identification System Inc.). The grading probe was inserted perpendicularly at the grading site between the 3rd and 4th last ribs, 7-cm off the split-line according to Canadian grading standards (Pomar and Marcoux, 2003). Backfat thickness and muscle depth (ie loin depth) measurements were used to obtain the predicted lean yield value for each carcass using the following equation (Bohrer et al. 2023b):

Destron predicted lean yield (2023 equation) = 89.16298—(1.63023 × backfat thickness)—(0.42126 × muscle depth) + (0.01930 × backfat thickness^2^) + (0.00308 × muscle depth^2^) + (0.00369 × backfat thickness × muscle depth).

Where backfat thickness (mm) and the muscle depth (mm) are collected at the grading site for each carcass.

### Carcass cutting yields

A total of 283 carcasses (target of 24 per treatment per marketing event) were randomly selected for carcass cutting yield evaluation. Carcasses were weighed after 20 to 24 h of chilling, and left sides were fabricated according to proprietary specifications of the processing facility. The shoulder was separated from the loin/belly between the fourth and fifth ribs, and the ham was separated 2.5 cm from the aitch bone. The loin was separated from the belly with a straight cut parallel to the backbone, ventral to the *longissimus* muscle.

Primal cuts were processed as follows: hams were skinned and trimmed; bellies were skinned, squared (teat line removed and flank end squared), and had ribs removed; butt and picnic shoulders were skinned, deboned, and trimmed. All cuts were weighed, and additional trim was recorded separately. Weights were expressed both as absolute values and as a percentage of side weight.

### Meat and fat quality analyses

Meat quality analyses were conducted on the same 283 carcasses used for carcass cutting tests. Ultimate pH was obtained immediately following fabrication (approximately 20 to 24 h postmortem) by inserting a glass-tipped probe attached to a portable pH meter into the *longissimus* muscle. The pH meter was calibrated with pH = 4.01 and pH = 7.00 buffer solutions that were stored at refrigerated temperatures (≤4°C) prior to use and the average of two different locations within the *longissimus* muscle were reported. Drip loss was assessed using the EZ cup method described previously by Rasmussen and Andersson (1996). Duplicate 25-mm diameter cores from the *longissimus* muscle of each carcass were weighed at 24-h and at 48-h postmortem after being placed within duplicate EZ drip loss containers (Sarstedt product no. 86.290; Sarstedt Ag & Co., Nümbrecht, Germany) to quantify drip loss over a 24 h period at the storage temperature of 4°C. The duplicate drip loss samples were averaged. Instrumental lightness (L*) color values of lean tissue were determined using a handheld colorimeter with D_65_ light source, an 11-mm aperture, and a 0° observer (Minolta CR-400; Minolta Corp., Osaka, Japan) on the ventral edge of loins following fabrication. The average of four different locations along the *longissimus* muscle were reported. Subjective color, marbling, and firmness were evaluated on the ventral surface near the center of the loins (10th rib location) according to standards published by the National Pork Producers Council (NPPC 1991, 1999).

Iodine value of subcutaneous fat samples was predicted using FT-NIR (Fourier Transform–Near Infrared) spectroscopy. Instrumental lightness (L*) color values of fat tissue were determined using a handheld colorimeter with D_65_ light source, an 11-mm aperture, and a 0° observer (Minolta CR-400; Minolta Corp., Osaka, Japan) on a fat sample from the surface of the trimmed belly. Immediately following fabrication, belly flop angle measurements were collected when trimmed bellies were suspended over a 2.5-cm bar at the midpoint between the most anterior and most posterior ends. Flop angle was determined at the bend site and the distance between the suspended sides of the belly was recorded 15-cm from the bar and between the most anterior and most posterior ends. Subjective firmness was recorded using a 1–4 scoring system where 1 indicated very firm and 4 indicated very soft. Fat percentage was determined on a center-cut slice from bellies using a low-field time-domain NMR analyzer (Bruker minispec), calibrated against ether-extractable fat, with samples analyzed in duplicate.

### Statistical analyses

The experimental design for this study resulted in two independent factors (interval between doses and interval between dose-2 and marketing), each with two different levels (ie 2 × 2 factorial design). Pen was considered as the experimental unit for live performance and carcass characteristics data and carcass was considered the experimental unit for carcass cutting yields and meat quality data. Live performance data were analyzed with PROC MIXED of SAS (v. 9.4, SAS Inst. Inc.; Cary, NC, USA) as repeated measures over time with fixed effects of interval between doses, marketing interval, and their interaction and random effects of replication and room. An unstructured covariance structure was selected based on the analysis of the fit statistics. A slice statement was used to detect statistical differences at each time point. Carcass characteristics data were analyzed with PROC MIXED of SAS with fixed effects of interval between doses, marketing interval, and their interaction and random effects of replication and room. Carcass cutting yields and meat quality data were analyzed with PROC MIXED of SAS with fixed effects of interval between doses, marketing interval, and their interaction and random effects of cutting day (which coincided with marketing event). Multiple comparisons tests were performed between interaction means at *P* ≤ 0.10, differences will be discussed at *P* ≤ 0.05, and tendencies will be discussed at *P* ≤ 0.10.

## Results

### Live performance

No significant interactions for the interval between doses and marketing interval were present for live weight, ADFI, ADG, or G: F (Table 3). There were two instances of statistical tendencies. For ADG from d 21 to 35 (interaction *P* = 0.07) – within the long marketing interval treatment, gilts with a 4-wk interval between doses had lower numerical ADG values compared with gilts with a 6-wk interval between doses; whereas within the short marketing interval treatment, gilts with a 4-wk interval between doses had greater numerical ADG values compared with gilts with a 6-wk interval between doses. For ADG from d 49 to 63 (interaction *P* = 0.10) – within the long marketing interval treatment, gilts with a 4-wk interval between doses had greater numerical ADG values compared with gilts with a 6-wk interval between doses; whereas within the short marketing interval treatment, gilts with a 4-wk interval between doses had lower numerical ADG values compared with gilts with a 6-wk interval between doses.

**Table 3:** The effect of dose timing schedule on live performance characteristics of gilts managed using GnRF immunization (whole population analysis).

	Treatment^1^^,^^2^					*P*-values		
*Marketing Interval -*	Long (7-wk weighted average)		Short (5-wk weighted average)		SEM^3^	Marketing Interval	Interval Between Doses	Interaction
*Interval Between Doses -*	4-wk	6-wk	4-wk	6-wk				
***Number of pens (pigs)^4^, no.***	12 (264)	12 (264)	12 (264)	12 (264)				
***Live weight by study day, kg***								
*** d 0***	31.6	31.7	31.8	31.6	0.46	0.82	0.97	0.70
*** d 21***	51.8	51.7	52.4	51.7	0.68	0.37	0.47	0.41
*** d 35***	64.3	64.8	65.8	64.7	0.75	0.24	0.62	0.18
*** d 49***	77.0	77.8	78.5	77.8	0.88	0.19	0.87	0.19
*** d 63***	93.6	93.8	92.3	92.3	1.37	0.07	0.96	0.89
*** d 85***	114.3	115.7	113.4	113.6	1.90	0.06	0.30	0.46
*** Live weight at marketing^5^***	126.2	127.6	125.6	127.1	2.12	0.54	0.11	0.93
***Average daily feed intake, kg/day***								
*** d 0 to 21***	1.91	1.91	1.92	1.87	0.041	0.51	0.29	0.42
*** d 21 to 35***	2.16	2.19	2.21	2.16	0.046	0.63	0.76	0.27
*** d 35 to 49***	2.22	2.24	2.26	2.28	0.066	0.12	0.40	0.94
*** d 49 to 63***	2.81^a^	2.83^a^	2.61^b^	2.65^b^	0.044	<0.01	0.54	0.76
*** d 63 to 85***	3.28^a^	3.26^a^	2.99^b^	3.01^b^	0.107	<0.01	0.95	0.69
*** d 85 to 105***	3.33	3.39	3.43	3.54	0.094	0.14	0.32	0.75
*** Overall period (d 0 to 105)***	2.53^a^	2.53^a^	2.46^b^	2.48^b^	0.034	0.02	0.76	0.81
***Average daily gain, kg/day***								
*** d 0 to 21***	0.97	0.97	0.99	0.97	0.016	0.32	0.16	0.31
*** d 21 to 35***	0.89	0.93	0.94	0.92	0.019	0.26	0.50	0.07
*** d 35 to 49***	0.90	0.91	0.92	0.93	0.019	0.28	0.38	0.89
*** d 49 to 63***	1.11^a^	1.07^a^	0.95^b^	0.98^b^	0.023	<0.01	0.85	0.10
*** d 63 to 85***	1.00	1.04	1.00	1.00	0.052	0.47	0.39	0.52
*** d 85 to 105***	0.79	0.79	0.84	0.87	0.050	0.12	0.70	0.79
*** Overall period (d 0 to 105)***	0.92	0.93	0.92	0.92	0.018	0.40	0.49	0.83
***Gain: Feed, kg/kg***								
*** d 0 to 21***	0.509	0.507	0.519	0.518	0.0074	0.14	0.83	0.97
*** d 21 to 35***	0.410	0.427	0.425	0.426	0.0083	0.25	0.13	0.21
*** d 35 to 49***	0.408	0.409	0.406	0.408	0.0087	0.82	0.79	0.91
*** d 49 to 63***	0.396^a^	0.379^ab^	0.365^b^	0.373^ab^	0.0079	0.02	0.57	0.14
*** d 63 to 85***	0.306^b^	0.319^ab^	0.332^a^	0.334^a^	0.0088	<0.01	0.20	0.37
*** d 85 to 105***	0.238	0.234	0.245	0.245	0.0157	0.31	0.80	0.85
*** Overall period (d 0 to 105)***	0.365	0.367	0.372	0.372	0.0047	0.03	0.73	0.60

ab Least squares means within each row with different superscripts are significantly different (*P* ˂ 0.05).

1 The interval between doses of the GnRF immunization was either 4 or 6 wks and the marketing interval was either a weighted average of 49 days (Long) or a weighted average of 35 days (Short).

2 Pigs were marketed in three groups on wk-12, wk-14, and wk-15 of the study; the heaviest 3–5 pigs in each pen (approximately 18%) were marketed on wk-12 and wk-14, and the barn dump (approximately 64%) occurred on wk-15.

3 SEM = standard error of the mean.

4 This was the number of gilts that started the study (ie does not account for removals or missing datapoints).

5 Weight of all pigs at marketing were recorded (at wk-12 first marketing group, at wk-14 second marketing group, and at wk-15 final marketing group).

There were no significant main effects (*P* > 0.10) for the interval between doses for live weight, ADFI, ADG, or G: F. There were several significant main effects for marketing interval. Gilts with the long marketing interval tended to be heavier on d 63 (*P* = 0.07) and on d 85 (*P* = 0.06) compared with gilts with the short marketing interval; however, there was no difference among marketing interval treatments for live weight at marketing (*P* = 0.54). Gilts with the long marketing interval had greater ADFI during d 49 to 63 (*P* < 0.01), during d 63 to 85 (*P* < 0.01), and for the overall study period (*P* = 0.02) compared with gilts with the short marketing interval. Gilts with the long marketing interval had greater ADG during d 49 to 63 (*P* < 0.01) compared with gilts with the short marketing interval; however, there was no difference among the marketing interval treatments for ADG during the overall study period (*P* = 0.40). Gilts with the long marketing interval had greater G: F during d 49 to 63 (*P* = 0.02) and had poorer G: F during d 63 to 85 (*P* < 0.01) compared with gilts with the short marketing interval. This resulted in gilts with the long marketing interval having poorer G: F during the overall study period (*P* = 0.03) compared with gilts with the short marketing interval.

### Carcass characteristics

No significant interactions for the interval between doses and marketing interval were present for HCW, carcass dressing percentage, or muscle depth (Table 4). There were two instances of statistical tendencies, which were backfat thickness (*P* = 0.09) and predicted lean yield (*P* = 0.08). Within the long marketing interval treatment, gilts with a 4-wk interval between doses had greater backfat thickness and lower predicted lean yield compared with gilts with a 6-wk interval between doses; whereas within the short marketing interval treatment, gilts with a 4-wk interval between doses had less backfat thickness and greater predicted lean yield compared with gilts with a 6-wk interval between doses.

**Table 4:** The effect of dose timing schedule on carcass characteristics of gilts managed using GnRF immunization (whole population analysis).

	Treatment^1^^,^^2^					*P*-values		
*Marketing Interval -*	Long (7-wk weighted average)		Short (5-wk weighted average)		SEM^3^	Marketing Interval	Interval Between Doses	Interaction
*Interval Between Doses -*	4-wk	6-wk	4-wk	6-wk				
***Number of pens (pigs)^4^, no.***	12 (229)	12 (219)	12 (231)	12 (227)				
***Hot carcass weight, kg***	101.3	102.2	100.6	102.5	1.13	0.67	0.01	0.39
***Carcass dressing percentage, %***	80.32	80.20	80.18	80.67	0.640	0.71	0.69	0.49
***Backfat thickness, mm***	18.3^a^	17.9^ab^	17.1^b^	17.6^ab^	0.27	<0.01	0.99	0.09
***Muscle depth, mm***	62.9^b^	64.2^ab^	63.5^ab^	64.6^a^	0.52	0.24	0.01	0.82
***Destron predicted lean yield (2023 equation)^5^, %***	56.13^b^	56.43^ab^	56.98^a^	56.67^ab^	0.191	<0.01	0.98	0.08

ab Least squares means within each row with different superscripts are significantly different (*P* ˂ 0.05).

1 The interval between doses of the GnRF immunization was either 4 or 6 wks and the marketing interval was either a weighted average of 49 days (Long) or a weighted average of 35 days (Short).

2 Pigs were marketed in three groups on wk-12, wk-14, and wk-15 of the study; the heaviest 3–5 pigs in each pen (approximately 18%) were marketed on wk-12 and wk-14, and the barn dump (approximately 64%) occurred on wk-15.

3 SEM = standard error of the mean.

4 This was the number of carcasses that data were collected for (excludes removed pigs and missing data points).

5 The equation used for Destron predicted lean yield (2023 equation) was the following: = 89.16298 – (1.63023 × backfat thickness) – (0.42126 × muscle depth) + (0.01930 × backfat thickness^2^) + (0.00308 × muscle depth^2^) + (0.00369 × backfat thickness × muscle depth).

There were significant main effects for the interval between doses for HCW and muscle depth. Gilts with a 4-wk interval between doses had lighter HCW (*P* = 0.01) and less muscle depth (*P* = 0.01) compared with gilts with a 6-wk interval between doses. There were no significant main effects (*P* > 0.10) for the interval between doses for carcass dressing percentage, backfat thickness, or predicted lean yield.

There were significant main effects for marketing interval for backfat thickness and predicted lean yield. Gilts with the long marketing interval had greater backfat thickness (*P* < 0.01) and less predicted lean yield (*P* < 0.01) compared with gilts with the short marketing interval. There were no significant main effects (*P* > 0.10) for the marketing interval for HCW, carcass dressing percentage, or muscle depth.

### Carcass cutting yields

To avoid redundancy, cut weights expressed as a percentage of side weight will be discussed rather than the raw weights. No significant interactions for the interval between doses and marketing interval were present for carcass cutting yields (Table 5).

**Table 5:** The effect of dose timing schedule on carcass cutting yields of gilts managed using GnRF immunization (subpopulation analysis).

	Treatment^1^^,^^2^					*P*-values		
*Marketing Interval -*	Long (7-wk weighted average)		Short (5-wk weighted average)		SEM^3^	Marketing Interval	Interval Between Doses	Interaction
*Interval Between Doses -*	4-wk	6-wk	4-wk	6-wk				
***Number of carcasses^4^, no.***	71	72	70	70				
***Hot carcass weight, kg***	102.4	103.0	102.1	101.8	1.07	0.12	0.67	0.32
***Cold carcass weight, kg***	96.1	96.5	96.1	94.9	1.06	0.16	0.43	0.16
***Cooler shrink loss, %***	6.11	6.40	5.86	6.79	0.355	0.84	0.08	0.36
***Trimmed ham weight, kg***	9.24	9.31	9.36	9.27	0.134	0.53	0.93	0.27
***Trimmed ham, expressed as a percentage of side weight, %***	19.25	19.34	19.49	19.59	0.161	0.09	0.50	0.99
***Trimmed loin weight, kg***	8.61	8.76	8.66	8.72	0.212	0.92	0.18	0.55
***Trimmed loin, expressed as a percentage of side weight, %***	17.93	18.18	18.04	18.41	0.394	0.30	0.06	0.70
***Trimmed belly weight, kg***	4.69^a^	4.70^a^	4.58^b^	4.55^b^	0.080	0.01	0.78	0.76
***Trimmed belly, expressed as a percentage of side weight, %***	9.77^a^	9.74^a^	9.52^b^	9.58^b^	0.164	0.03	0.89	0.64
***Side ribs weight, kg***	1.58^a^	1.55^ab^	1.54^ab^	1.49^b^	0.031	<0.01	0.01	0.72
***Side ribs, expressed as a percentage of side weight, %***	3.29^a^	3.21^b^	3.20^b^	3.15^ab^	0.064	0.02	0.03	0.66
***Trimmed butt shoulder weight, kg***	2.22	2.21	2.19	2.17	0.032	0.14	0.49	0.78
***Trimmed butt shoulder, expressed as a percentage of side weight, %***	4.61	4.58	4.55	4.57	0.068	0.39	0.84	0.58
***Trimmed picnic shoulder weight, kg***	5.97	6.03	5.96	5.93	0.068	0.19	0.72	0.31
***Trimmed picnic shoulder, expressed as a percentage of side weight, %***	12.43	12.52	12.40	12.50	0.122	0.73	0.21	0.98
***Trimmed primals weight^5^, kg***	32.30	32.54	32.28	32.12	0.408	0.22	0.82	0.27
***Trimmed primals, expressed as a percentage of side weight, %***	67.27	67.56	67.20	67.77	0.601	0.82	0.16	0.64
***Additional trim weight, kg***	9.77	9.77	9.54	9.45	0.427	0.02	0.73	0.68
***Additional trimmings, expressed as a percentage of side weight***	20.30	20.24	19.82	19.92	0.780	0.08	0.93	0.72

ab Least squares means within each row with different superscripts are significantly different (*P* ˂ 0.05).

1 The interval between doses of the GnRF immunization was either 4 or 6 wks and the marketing interval was either a weighted average of 49 days (Long) or a weighted average of 35 days (Short).

2 Pigs were marketed in three groups on wk-12, wk-14, and wk-15 of the study; the heaviest 3–5 pigs in each pen (approximately 18%) were marketed on wk-12 and wk-14, and the barn dump (approximately 64%) occurred on wk-15.

3 SEM = standard error of the mean.

4 This was the number of carcasses that were used for this portion of the study.

5 Trimmed primals were calculated as the sum of the trimmed ham, the trimmed loin, the trimmed belly, the side ribs, the trimmed butt shoulder, and the trimmed picnic shoulder.

There were several significant main effects for the interval between doses. Gilts with a 4-wk interval between doses tended to have less cooler shrink loss (*P* = 0.08), tended to have a lower percentage of trimmed loin (*P* = 0.06), and had a greater percentage of side ribs (*P* = 0.03) compared with gilts with a 6-wk interval between doses. There were no significant main effects (*P* > 0.10) for the interval between doses for carcass side weight (of the selected subpopulation of pigs), trimmed ham percentage, trimmed belly percentage, trimmed butt shoulder percentage, trimmed picnic shoulder percentage, or additional trim percentage.

There were several significant main effects for marketing interval. Gilts with a long marketing interval tended to have a lower percentage of trimmed ham (*P* = 0.09), had a greater percentage of trimmed belly (*P* = 0.03), had a greater percentage of side ribs (*P* = 0.02), and tended to have a greater percentage of trim (*P* = 0.08) compared with gilts with a short marketing interval. There were no significant main effects (*P* > 0.10) for the marketing interval for carcass side weight (of the selected subpopulation of pigs), cooler shrink loss, trimmed loin percentage, trimmed butt shoulder percentage, and trimmed picnic shoulder percentage.

### Loin quality

No significant interactions for the interval between doses and marketing interval were present for loin quality traits (Table 6). Additionally, there were no significant main effects for the interval between doses for loin quality traits. There were no significant main effects for the marketing interval for loin quality traits, with the lone exception of NPPC firmness, which was lower (*P* = 0.03) for gilts with the long marketing interval compared with gilts with the short marketing interval.

**Table 6:** The effect of dose timing schedule on loin quality of gilts managed using GnRF immunization (subpopulation analysis).

	Treatment^1^^,^^2^^,^^3^^,^^4^					*P*-values		
*Marketing Interval -*	Long (7-wk weighted average)		Short (5-wk weighted average)		SEM^3^	Marketing Interval	Interval Between Doses	Interaction
*Interval Between Doses -*	4-wk	6-wk	4-wk	6-wk				
***Number of carcasses^4^, no.***	71	72	70	70				
***Ultimate pH***	5.62	5.62	5.65	5.61	0.024	0.39	0.26	0.24
***Drip loss, %***	1.48	1.53	1.64	1.52	0.186	0.65	0.84	0.57
Minolta L*	46.40	46.33	46.08	46.30	0.358	0.45	0.74	0.53
***NPPC color***	3.18	3.08	3.20	3.10	0.108	0.72	0.11	0.99
***NPPC marbling***	2.24	2.20	2.18	2.08	0.143	0.28	0.39	0.75
***NPPC firmness***	1.23	1.19	1.35	1.30	0.055	0.03	0.41	0.92

1 The interval between doses of the GnRF immunization was either 4 or 6 wks and the marketing interval was either a weighted average of 49 days (Long) or a weighted average of 35 days (Short).

2 Pigs were marketed in three groups on wk-12, wk-14, and wk-15 of the study; the heaviest 3–5 pigs in each pen (approximately 18%) were marketed on wk-12 and wk-14, and the barn dump (approximately 64%) occurred on wk-15.

3 SEM = standard error of the mean.

4 This was the number of carcasses that were used for this portion of the study.

### Fat quality and belly firmness

There were several significant interactions for the interval between doses and marketing interval for fat quality and belly firmness (Table 7). There was an interaction for the interval between doses and marketing interval for belly flop angle (*P* = 0.03) and there were two instances of statistical tendencies, which were the two belly flop distance measurements [at 15 cm from the bar (*P* = 0.09) and when measured from the edges of the belly (*P* = 0.08)]. Each of these significant interactions were caused by greater levels of belly firmness observed for gilts with the 6-wk interval between doses compared with gilts with the 4-wk interval between doses in the short marketing interval treatments; whereas, minimal differences were observed for the interval between doses treatments in the long marketing interval treatments.

**Table 7:** The effect of dose timing schedule on fat quality and belly firmness of gilts managed using GnRF immunization (subpopulation analysis).

	Treatment^1^^,^2^,^3^,^4^^					*P*-values		
*Marketing Interval -*	Long (7-wk weighted average)		Short (5-wk weighted average)		SEM^3^	Marketing Interval	Interval Between Doses	Interaction
*Interval Between Doses -*	4-wk	6-wk	4-wk	6-wk				
***Number of carcasses^4^, no.***	71	72	70	70				
***Iodine value***	68.1^b^	68.1^b^	69.4^a^	69.4^a^	0.53	<0.01	0.88	0.96
***Belly flop angle,°^5^***	69.0^a^	68.1^a^	53.7^b^	64.0^a^	5.42	<0.01	0.07	0.03
***Belly flop distance (15 cm from bar)^5^, mm***	27.4^a^	27.4^a^	21.8^b^	25.3^a^	2.00	<0.01	0.10	0.09
***Belly flop distance (edges of belly)^5^, mm***	31.2^a^	31.6^a^	26.1^b^	30.0^a^	2.02	<0.01	0.04	0.08
***Belly subjective firmness^5^***	1.36	1.50	1.66	1.67	0.078	<0.01	0.30	0.36
***Belly slice fat composition, %***	39.97	40.36	40.13	39.53	0.863	0.47	0.82	0.29

ab Least squares means within each row with different superscripts are significantly different (*P* ˂ 0.05).

1 The interval between doses of the GnRF immunization was either 4 or 6 wks and the marketing interval was either a weighted average of 49 days (Long) or a weighted average of 35 days (Short).

2 Pigs were marketed in three groups on wk-12, wk-14, and wk-15 of the study; the heaviest 3–5 pigs in each pen (approximately 18%) were marketed on wk-12 and wk-14, and the barn dump (approximately 64%) occurred on wk-15.

3 SEM = standard error of the mean.

4 This was the number of carcasses that were used for this portion of the study.

5 Temperature served as a covariate for belly flop and firmness measurements.

There were several significant main effects for the interval between doses for fat quality and belly firmness traits. Gilts with a 4-wk interval between doses tended to have lower belly flop angle (*P* = 0.07), tended to have less belly flop distance 15 cm from the bar (*P* = 0.10), and had less belly flop distance when measured at the edges of the belly (*P* = 0.04) compared with gilts with a 6-wk interval between doses. There were not significant main effects for the interval between doses for iodine value, subjective belly firmness, or belly slice fat composition.

There were several significant main effects for the marketing interval for fat quality and belly firmness traits. Gilts with long marketing interval had lower iodine value (*P* < 0.01), greater belly flop angle (*P* < 0.01), greater belly flop distance 15 cm from the bar (*P* < 0.01), greater belly flop distance when measured at the edges of the belly (*P* < 0.01), and lower subjective belly firmness scores (*P* < 0.01) compared with gilts with short marketing interval. There was not a significant main effect for marketing interval for belly slice fat composition.

## Discussion

It was hypothesized that changes in the interval between doses and in the marketing interval after dose-2 would impact production traits of gilts with GnRF immunization. The underlying premise of this hypothesis was that the physiological response to GnRF immunization is time-dependent, and therefore, deviations in the timing of dose schedules could result in measurable changes in growth performance, feed efficiency, and carcass composition. By evaluating both dosing intervals simultaneously, this study aimed to better define the production consequences associated with flexibility in dose timing, which is increasingly relevant in commercial production systems where logistical and labor constraints may necessitate adjustments to on-farm schedules.

To our knowledge, this experiment is the first controlled evaluation of how different dosing intervals affect production characteristics in pigs with GnRF immunization. Previous research in this area has largely focused on the physiological and production responses following administration of dose-2, with limited attention given to the potential implications of extending or shortening the interval between the initial priming dose and the subsequent booster dose. There was speculation prior to the initiation of this study that extending the interval between doses could lead to degradation or attenuation of circulating antibody titers prior to administration of dose-2. This speculation was supported by previous research demonstrating anti-GnRF antibody reduction as the interval between doses increases (Dalmau et al. 2015; Genís et al. 2024). Such degradation could theoretically reduce the magnitude or consistency of the immunological response following dose-2, thereby diminishing the expected growth performance benefits associated with suppression of gonadal activity. This concern is particularly relevant given that GnRF immunization relies on the development and maintenance of sufficient antibody titers to effectively suppress the secretion of reproductive hormones (Allison et al. 2021; Genís et al. 2024). In contrast to these concerns, results from this study suggest that extending the interval between doses from 4 wk to 6 wk had minimal impact on growth performance, feed intake, or carcass traits of gilts. From a practical standpoint, these results suggest some flexibility in scheduling dose-2. However, because the inter-dose interval is confounded with age in the current study, this interpretation should be viewed with caution as age-related effects cannot be clearly distinguished from interval effects.

The interval between dose-2 and marketing (ie marketing interval) exerted a more pronounced influence on production and carcass characteristics. Several previous studies conducted in male pigs with GnRF immunization (Tavárez et al. 2016; Harris et al. 2017; Bohrer et al. 2024a) have consistently reported reductions in feed conversion efficiency and increased fat deposition as the marketing interval is extended. Similar trends have also been observed in female pigs with GnRF immunization (Vasquez-Hidalgo et al. 2023; Bohrer et al. 2024b; Kotatha et al. 2025), indicating that fundamental shifts in nutrient utilization following prolonged suppression of gonadal function may occur in both male and female pigs. Collectively, these studies indicate that the effects of GnRF immunization on growth performance are both sex- and physiology-dependent. In males, any performance advantages are largely attributable to the period prior to the second dose when compared with physically castrated barrows. Following the second dose, the post-immunization state is characterized by increased feed intake and reduced feed efficiency. In contrast, with gilts, suppression of gonadal function after the second dose can increase feed intake and late-finishing gain and promote greater levels of fat deposition when compared with non-immunized gilts. However, prolonged exposure to this state may also reduce feed efficiency and negatively alter carcass outcomes. Data from this study suggest meaningful production impacts were found between gilts with a long marketing interval (7-wk weighted average) and gilts with a short marketing interval (5-wk weighted average). During the grow-finish period, gilts with a long marketing interval had 2.4% greater ADFI and similar ADG compared with gilts with a short marketing interval–resulting in a 1.6% reduction in G: F. These changes in growth performance were accompanied by notable differences in carcass composition and quality. Gilts with the long marketing interval had 0.8 mm greater backfat thickness and a 0.55% reduction in predicted lean yield compared with gilts with a short marketing interval, suggesting a redistribution of nutrients toward adipose tissue deposition. In addition, carcass fabrication data revealed a 0.20% increase in trimmed belly weight expressed as a percentage of side weight. Changes in lipid composition were also observed, as evidenced by a 1.3-unit reduction in iodine value, along with greater subjective and objective indications of belly firmness in gilts with the long marketing interval compared with gilts with the short marketing interval. The carcass and meat quality responses are consistent with increased fat accretion and altered fatty acid composition associated with prolonged suppression of ovarian activity (Vasquez-Hidalgo et al. 2023; Bohrer et al. 2024b; Kotatha et al. 2025).

From a practical perspective, these findings highlight a key trade-off when determining the optimal marketing interval for gilts with GnRF immunization, as extended intervals may increase weight gain and fat deposition but reduce feed efficiency. Given that increased fat deposition can be economically advantageous for packers, particularly when associated with greater belly yield and lower iodine value, balancing efficiency with carcass quality is essential. From a physiological standpoint, the results of this study align with established expectations following suppression of ovarian activity in gilts. Previous research has demonstrated that suppression of ovarian function leads to increased voluntary feed intake and weight gain (Bohrer et al. 2014; Poulsen Nautrup et al. 2020). However, when the marketing interval is extended, the efficiency with which nutrients are converted into lean tissue growth appears to decline. Instead, nutrient partitioning shifts progressively toward fat deposition, resulting in reduced feed efficiency and altered carcass composition (Vasquez-Hidalgo et al. 2023; Bohrer et al. 2024b; Kotatha et al. 2025).

## Conclusion

The primary finding of this study was that the time between dose-2 and marketing exerted a far greater influence on live performance, carcass composition, and fat quality than the interval between doses. Collectively, these findings underscore a clear trade-off–shorter marketing interval favors feed efficiency and lean yield, whereas longer marketing interval enhances fat firmness and belly quality. Therefore, optimal dose timing strategies for GnRF-immunized gilts should be tailored to the relative economic value of feed efficiency versus carcass fat quality within a given production and marketing system.

## Abbreviations

ADFI: average daily feed intake
ADG: average daily gain
G:F: gain:feed
GnRF: gonadotropin-releasing factor
HCW: Hot carcass weight
NPPC: National Pork Producers Council
